# Measles Virus Infection Fosters Dendritic Cell Motility in a 3D Environment to Enhance Transmission to Target Cells in the Respiratory Epithelium

**DOI:** 10.3389/fimmu.2019.01294

**Published:** 2019-06-05

**Authors:** Shaghayegh Derakhshani, Andreas Kurz, Lukasz Japtok, Fabian Schumacher, Lisa Pilgram, Maria Steinke, Burkhard Kleuser, Markus Sauer, Sibylle Schneider-Schaulies, Elita Avota

**Affiliations:** ^1^Institute for Virology and Immunobiology, University of Wuerzburg, Wuerzburg, Germany; ^2^Department for Biotechnology and Biophysics, University of Wuerzburg, Wuerzburg, Germany; ^3^Department of Toxicology, Institute of Nutritional Science, University of Potsdam, Nuthetal, Germany; ^4^Department of Molecular Biology, University of Duisburg-Essen, Essen, Germany; ^5^Fraunhofer Institute for Silicate Research ISC, Chair of Tissue Engineering and Regenerative Medicine, University Hospital Wuerzburg, University of Wuerzburg, Wuerzburg, Germany

**Keywords:** dendritic cell, cell migration, measles virus, 3D tissue model, sphingosine-1-phosphate

## Abstract

Transmission of measles virus (MV) from dendritic to airway epithelial cells is considered as crucial to viral spread late in infection. Therefore, pathways and effectors governing this process are promising targets for intervention. To identify these, we established a 3D respiratory tract model where MV transmission by infected dendritic cells (DCs) relied on the presence of nectin-4 on H358 lung epithelial cells. Access to recipient cells is an important prerequisite for transmission, and we therefore analyzed migration of MV-exposed DC cultures within the model. Surprisingly, enhanced motility toward the epithelial layer was observed for MV-infected DCs as compared to their uninfected siblings. This occurred independently of factors released from H358 cells indicating that MV infection triggered cytoskeletal remodeling associated with DC polarization enforced velocity. Accordingly, the latter was also observed for MV-infected DCs in collagen matrices and was particularly sensitive to ROCK inhibition indicating infected DCs preferentially employed the amoeboid migration mode. This was also implicated by loss of podosomes and reduced filopodial activity both of which were retained in MV-exposed uninfected DCs. Evidently, sphingosine kinase (SphK) and sphingosine-1-phosphate (S1P) as produced in response to virus-infection in DCs contributed to enhanced velocity because this was abrogated upon inhibition of sphingosine kinase activity. These findings indicate that MV infection promotes a push-and-squeeze fast amoeboid migration mode via the SphK/S1P system characterized by loss of filopodia and podosome dissolution. Consequently, this enables rapid trafficking of virus toward epithelial cells during viral exit.

## Introduction

The ability of DCs to migrate through tissues is essential for an effective immune response. Their ability to transit within and from peripheral to lymphoid compartments is abused by certain pathogens which target DCs to access sites where they are amplified and spread systemically (1–3). In contrast, their importance for late stages of viral replication by transmitting virus to peripheral sites for subsequent release from these compartments is less well-documented.

Because it is very contagious [basic reproduction rate (R_0_ = 12–18) (4)] and the infection-associated disease morbidity/mortality rates continue to be high, measles virus (MV) is a clinically highly relevant pathogen. As suggested by *in vitro* and *ex vivo* studies, its interaction with DCs may be central to MV pathogenesis (5–7). In the early phase of infection and systemic spread, MV targets cells of the lympho/monocytic lineage which express its entry receptor CD150. In addition to macrophages, DCs rather than respiratory tract epithelial cells are prime early targets which serve as transport vehicles into the secondary lymphatic tissues to initiate viral transmission to lymphoid cells (8–10). In contrast to the early phase, MV infection of respiratory tract epithelial cells is apparent at late infection stages: then nectin-4, expressed on their basolateral surface as receptor becomes accessible to the virus (11, 12), and this is essential for efficient viral exit from this compartment and horizontal transmission (13, 14). Detection of infected DCs and infectious foci both in humans and experimentally infected macaques in close proximity to the respiratory tract epithelium suggested their function as vehicles transmitting MV to epithelial cells (13, 15–18). When applied to the basolateral surface of lung epithelial cells, infected B lymphoblastoid or myeloid cells (also including monocyte-derived DCs, Mo-DCs) efficiently transmitted MV *in vitro*, which relied on nectin-4 on the target cells (11, 14, 19, 20). Formation of organized virological synapses between MV-infected DCs and T cells as important for transmission has been described, while transmission interfaces between MV-infected DCs and epithelial cells are less well-characterized (3, 21). Though these studies suggested that infected DCs could serve as important viral donors both early and late in infection, their experimental setup did not allow to address the impact of MV-infection on DC migration which is of crucial importance and prerequisite for successful interaction with target cells and transmission.

Studies on viability, function and chemotactic responses of MV-infected DCs (MV-DC) *in vitro* have so far relied on 2D cultures also involving co-cultured acceptor cells, yet fail to integrate micro-environmental conditions these cells are exposed to in a complex tissue (6, 22–24). There, communication with tissue resident cells and transduction of contractile cytoskeletal to mechanical forces during locomotion may substantially impact transmission efficiency. Detailed information of factors promoting tissue motility of infected DCs and MV cell-cell transmission in the respiratory tract late in infection would be of obvious importance in development of interventive regimen for viral exit and transmission.

This cannot be addressed using intravital microscopy in mice as successfully employed in other infection models [recently reviewed in (25)] because mice are not permissive for peripheral MV infection. Therefore, surrogate complex human culture methods recapitulating distinct tissue features are needed that allow for 3D visualization and robust quantitative analysis and thereby, pivotal information on spatial and temporal features of host cell-pathogen interactions.

Based on our previously published data we generated human 3D airway mucosa tissue models consisting of a small intestinal submucosa (SIS) scaffold with embedded primary human fibroblasts and H358 lung epithelial cells (26). Whereas, airway tissue models generated on transwell inserts allow to study cell migration through synthetic porous membranes (27), our tissue models mimic the respiratory mucosa and, thus, facilitate investigations on cell migration through fibroblast-loaded *in vivo*-like connective tissue. To evaluate the impact of MV-infection on DC motility and consequences for viral transmission to lung epithelial cells, we advanced our tissue model by including human primary DCs. As reported for transwell-based systems, Mo-DCs added basolateral migrated toward the epithelial layer (27, 28).

In our model system, added infected DC cultures were effective at mediating MV epithelial cell infection in a nectin-4-dependent manner, indicating that motility, formation of transmission structures and receptor usage were retained. Surprisingly, motility both with regard to velocity and accumulated distance was enforced for MV-infected DCs as compared to their uninfected siblings indicating that MV infection promoted cytoskeletal activity in DCs. This was directly reflected by morphological polarization, and occurred independently of factors released from epithelial or fibroblast cells. Using specific inhibitors, we established that the infection-regulated sphingosine kinase (SphK)/S1P system contributed to enhancement of infected DC migration in 3D which was amoeboid rather than mesenchymal-like as revealed by reduction of filopodial activity and detectable podosome structures.

## Materials and Methods

### Ethics Statement

Primary human immune cells were obtained from blood of healthy donors at Department of Transfusion Medicine, University of Wuerzburg. Human primary fibroblasts were isolated from skin biopsies. Written informed consent was obtained from the participants of this study. All experiments involving human material were conducted according to the principles of the Declaration of Helsinki and ethically approved by the ethical committee of the Medical Faculty of the University of Wuerzburg.

### Cells and Generation of Monocyte-Derived Dendritic Cells

Human epithelial lung adenocarcinoma H358 cells (ATCC® CRL-5807™, Manassas, USA) were cultured in RPMI 1640 supplemented with 10% FCS. H358 nectin-4 knockout cells were generated using CRISPR-Cas9 based genetic manipulation using a lentiviral system. Retroviral particles transferring Cas9 (retroviral transduction vector, pRSGCCH-U6-sg-HTS6C-CMV-Cas9-2A-Hygro, Cellecta, California, USA) and thereafter nectin-4 sgRNA (lentiviral sgRNA, GSGH11838-246559205, Dharmacon, Cambridge, UK) to H358 cells were generated by transfection of HEK-293, as described before (29, 30). Human primary fibroblasts were isolated from skin biopsies and cultured in DMEM-10% FCS. DCs were generated from monocytes isolated from peripheral blood from healthy donors by culture with GM-CSF (500 U/ml, Berlex, Germany)/IL-4 (250 U/ml, Miltenyl Biotec, Germany) in RPMI 1640 containing 10% FCS for 5 to 6 days. Genetically tagged [MV IC323-GFP (31)] or untagged (for lipidomics) MV wildtype viruses were grown on Vero-hSLAM or lymphoblastoid BJAB cells, respectively, in RPMI 1640–10% FCS and used for a 24 h infection of DCs at a multiplicity of infection (MOI) of 2 with fusion inhibitory peptide (Z-D-Phe-L-Phe-Gly-OH; Bachem; 200 μM in DMSO) added immediately after infection to prevent spread and syncytium formation. Infection levels were determined by detection of GFP (tagged virus) or MV N protein (untagged virus; using an N protein specific monoclonal mouse antibody followed by a FITC conjugated secondary antibody) by flow cytometry after 24 h and ranged between 10 and 60%.

### Establishment and Characterization of Human Airway Infection 3D Models

3D human airway mucosa tissue models were generated based on a previously published procedure (26). Briefly, decellularized porcine SIS was used as a biological scaffold which was seeded with primary human fibroblasts from the apical side (50,000 cells/cm^2^) and after 24 h with H358 epithelial cells pre-labeled with cell proliferation dye eFluor 670 (eBioscience, San Diego, CA) (200,000 cells/cm^2^). Epithelial barrier integrity was daily analyzed by measuring the transepithelial electrical resistance (TEER) (Millicell®-ERS) for the 10 days of culture (indicating 900 ± 250 Ω^*^cm^2^). Resistance values were stratified for a model containing only the SIS. Infection models were established by addition of MV-infected DC-cultures (MV-DC) (250,000 cells consisting of both GFP+ and GFP- cells; pre-labeled with life-DAPI (NucBlue, Life technologies, Carlsbad, CA) for 15 min at 37°C) to the basolateral side of the flipped models for 1 h which were subsequently reverted back to their original orientation. When indicated, 3D tissue models were fixed in 4% paraformaldehyde, embedded in paraffin, sectioned at 3 μm thickness, deparaffinated in xylene and rehydrated. Sections were subjected to hematoxylin/eosin or immunofluorescent staining performed by first antigen retrieval in a steamer at pH 6 followed by incubation with primary antibodies (α-vimentin (ab8069, Abcam, Cambridge, UK), α-E-cadherin (EP700Y, Abcam, Cambridge, UK), APC-conjugated E-cadherin (EP700Y, Abcam, CamBiolegend, San Diego, CA) for 1 h at room temperature or overnight at 4° C. Alexa Fluor 555 goat-α-mouse, Alexa Fluor 647 goat α-rabbit and Alexa Fluor 647 goat α-mouse (all Life technologies, Carlsbad, CA) were used as secondary antibodies. F-actin was labeled using phalloidin ATTO 643 (ATTO-TEC GmbH, Dortmund, Germany).

### Imaging the DC Transmigration in 3D Tissue Models

For imaging DC transmigration, tissue models were transferred 1 h after addition of MV-DCs onto confocal microscopy 35 mm diameter, high Glass Bottom μ-Dish (ibidi, Munich, Germany) and z-stacks through the entire construct were acquired. Live imaging was performed by an inverted microscope (CLSM 780; Zeiss, Germany) equipped with an incubation system. A time lapse z-stack was recorded using 20x apochromat for transmigration live imaging for 2 h. Image analysis for quantification and 3D cell tracking was performed using Imaris software (Bitplane).

### MV Transmission to Epithelial Cells

For analyzing the MV transmission to H358 epithelial cells, MV-infected tissue models were cultured for 3–4 days following back-flipping. Then, GFP+ cells were imaged at the apical model surface using an inverted fluorescence microscope (Leica DMi8, Germany). GFP+ areas in epithelial cells (eFluor 647 positive) were detected by intensity based segmentation. Infection levels were estimated according to intensity and the corresponding covered areas (data not shown).

For quantification, H358 cells were recovered from the models by trypsin /EDTA (0.5 M, 3 times, 5 min each at 37°C) and the frequencies of GFP+ epithelial cells were detected by flow cytometry. As H358 cells were not completely recovered from the models by this procedure, the GFP mean fluorescence intensity (MFI) levels determined by flow cytometry were compared with those obtained by intensity based image analysis (mentioned above) performed prior to flow cytometry on the same models in order to support the flow cytometry results.

### Morphological Analyses

MV-DC cultures were seeded for 2 h at 37°C onto fibronectin (FN)-coated ibidi channel slides (2 x 10^5^ cells/channel) (μ-Slide VI0.4 ibidi; 20 μg/mL fibronectin in PBS at 37°C, 2 h, Prospec, Ness Ziona, Israel), fixed in paraformaldehyde (4% in PBS), permeabilized (0.1% Triton X-100) and used for detection of F-actin by phalloidin ATTO 643 or podosomes (after a blocking step in BSA 5% in PBS) by an α-vinculin antibody (clone hVIN-1, Sigma-Aldrich, St. Louis, MI). Imaging was performed using an inverted confocal laser scanning microscope LSM 780 (Zeiss, Germany), equipped with a 63x or a 40x plan apochromat (NA 1.4, both oil immersion). Excitation wavelengths were 405, 488, 561, and 633 nm. Images were acquired and pre-processed using DAQ software ZEN2012 black.

Circularity analysis was carried out using ImageJ. As phalloidin based actin detection was not complete along the entire cell border, manual rather than automated segmentation was used to determine cell polarity. Cells close to the image borders, displaying more than one nucleus or lacing distinct cell borders in at least one fluorescence channel were discarded. Selected cell borders were measured and the circularity parameter was automatically calculated according to

(1) $$\begin{array}{r} \text{Circularity}=4\cdot \pi \cdot \left(\frac{area}{perimeter^{2}}\right) \end{array}$$

Circularity calculation yields values between 0 and 1, with 1 representing circular and values toward 0 representing a deformed elliptical shape.

### 3D Migration Assay

For cell tracking experiments, 1 × 10^6^ MV-DC cultures were resuspended in RPMI/10% FCS and loaded into un-polymerized gels consisting of fibrillar collagen matrices (2 mg/ml collagen I Rat Tail High Protein (Sigma Aldrich) adjusted to neutral pH with 7% bicarbonate and MEM into μ-Slide VI0.4 (ibidi, Munich, Germany). Tracks were recorded following collagen matrix polymerization at 37°C for 1 h. When indicated, DCs were pre-treated with Y27632 (30 μM, Cayman, Germany), SKI-II (10 μM, Sigma, Germany), or VPC (10 μM, Cayman, Germany) 2 h prior to life imaging. None of the inhibitors were toxic at the indicated concentrations. Cells were manually tracked using the Fiji plug in “Manual Tracking” followed by quantification of velocity and migration distances using the “Chemotaxis Tool” software.

### 3D Image Analysis

3D image analysis was performed using Imaris software (Bitplane). In order to quantify the number of DAPI+ cells (GFP+ or GFP-) in the measured volume we used the Imaris spot detection tool. Intensity point clouds were transformed into countable objects. The estimated diameter was set between 10–15 μm for the spot detection algorithm, detected spots were filtered with a quality parameter (Imaris). The values were chosen to achieve best overlap of detected spots with true fluorescence signal. Data was pre-processed with a median filter (3x3x1 px kernel) for better performance of the spot detection algorithm. For estimation of migration efficiency a ratio of cell counts in a top layer of DCs to the total DC count in the model was calculated. In order to keep the consistency of the analysis for different models, first we defined the distance between the z position of the lowest and highest GFP+ DC in the 3D model. Then the intermediated z position was calculated and the top layer was defined above the intermediate point. Dimensions of the representative model are 850, 850, 252 μm (X, Y, Z).

For tracking the cell movement expected to be random, an autoregressive motion algorithm was applied. Requirements for track registration were set with track length above 10 μm and track duration longer than 2.4 s to filter out of nonrecurring events. For the maximum distance between detected spot and predicted position in subsequent time points we set 13.9 μm and 26.5 μm for GFP+ or GFP-/DAPI+ cells. Dimensions of the representative model are 425, 425, 45 μm (X, Y, Z) with 3.5 min time intervals.

### Lipid Analysis

For lipid analysis 1 x 10^6^ DCs/sample (immature DCs exposed to MV (MOI 2) or equivalent amounts of a mock cell extract prepared from uninfected cells (MOCK) for 24 h) were dissolved in 1 mL methanol. S1P was extracted using C17-S1P (Avanti Polar Lipids, Alabaster, USA) as internal standard and quantified as described (32). Sample analysis was carried out by liquid chromatography tandem-mass spectrometry (LC-MS/MS) using a 6490 triple quadrupole mass spectrometer (Agilent Technologies, Waldbronn, Germany) operating in the positive electrospray ionization mode (ESI+).

### Statistical Analysis

Statistics were analyzed and graphs were prepared using GraphPad Prism 6. For comparing two groups, a two-tailed student's *t*-test was performed when having a normal distribution or the Mann-Whitney test when there was no normal distribution. For the statistical analysis the *P*-values are shown as ^*^P < 0.05, ^**^P < 0.01, ^***^P < 0.001, ^****^P < 0.0001 on graphs. Data shown was acquired in at least three independent experiments each consisting of at least one donor.

## Results

### MV Is Efficiently Transmitted to H358 Epithelial Cells by Infected DCs in a 3D Environment

**Figure 1:**
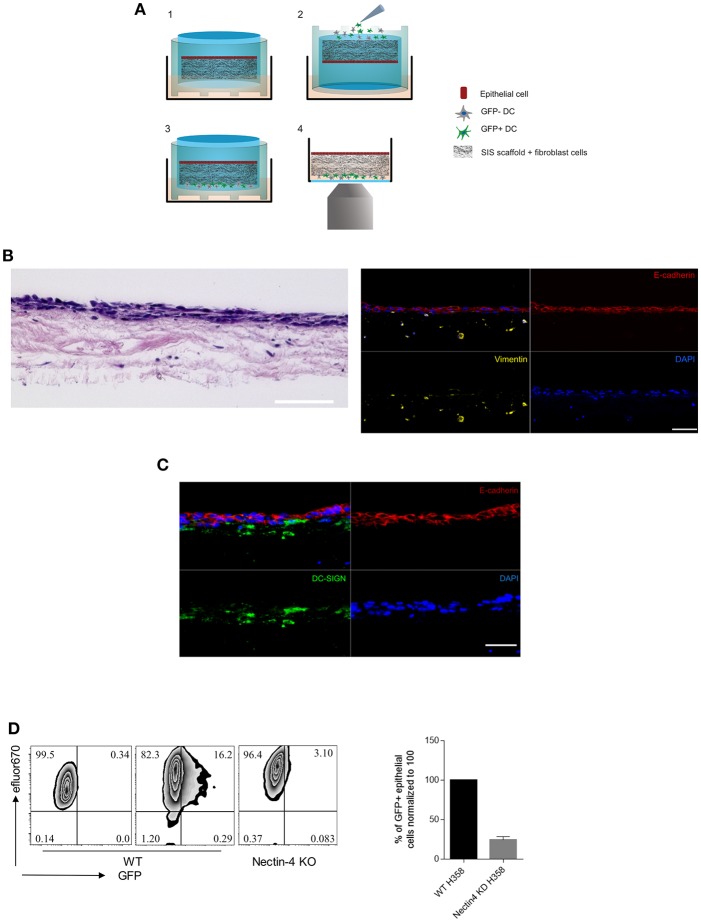
The 3D model system recapitulates MV transmission in the respiratory tract. **(A)** Schematic representation of (1) 3D model general structure, (2) flipping the model up-side-down to add the MV-DCs to the basolateral side, (3) reverting the model back to the original orientation after 1h. From this stage the model was incubated further for the fixation in time points of interest, or directly to (4) for life imaging experiments. **(B)** Hematoxylin/eosin (left) and immunofluorescent (IF) staining (right) for E-cadherin (apical epithelial cell layer, red), vimentin (collagen-embedded primary fibroblasts, yellow) and nuclei (DAPI, blue) of the 3D model tissue. **(C)** DC-SIGN+ cells (green) in close proximity to and within the epithelial layer (red) 24 h after basolateral addition of MV-exposed DCs. A, B: Scale bars, 100 μm. Overlays each in upper left panels**. (D)** MV transmission from MV-infected DCs to wild-type (middle graphs) and nectin-4 knockout H358 cells (right graph) was determined 4 days post addition of MV-DCs by flow cytometry. Uninfected wild type H358 cells were used as a negative control (left graph). H358 cells were labeled with eFluor 670 dye prior to addition to the model. The bar graph shows the mean ± SEM of MV infected epithelial cells (GFP+) percentage in three independent experiments each consisting three replicates.

To study parameters important in MV transmission to respiratory epithelial cells as occurring late in infection, we advanced our previously published 3D respiratory tract model. We seeded the decellularized porcine SIS with primary fibroblasts and H358 lung epithelial cells (Figure 1A) (26). Hematoxylin/eosin staining showed a dense cell multilayer on the apical surface of the tissue model and few cells that have migrated into the SIS scaffold. Immunofluorescent staining verified that E-cadherin-positive H358 built the epithelial layer whereas vimentin-positive fibroblasts migrated in the connective tissue (Figure 1B). Human peripheral blood monocytes were differentiated into immature DCs by culture in IL-4/GM-CSF (thereby precluding differentiation into macrophages). These are phenotypical and functional equivalents of the inflamed DC subset (Inf-DC) defined in mice in peripheral tissues also including the lung and distinct from cDC1 or cDC2 subsets differentiation of which occurs from the hematopoetic stem cell compartment and is Flt3-dependent (33–35). Immature mo-DCs were infected with MV-IC323-GFP for 24 h. Corroborating earlier studies by us and other laboratories, exposure to MV caused phenotypic maturation of the entire culture (22–24, 36–38) though DC infection levels varied donor-dependently (ranging from 10-60% as identified by GFP expression)(Figures S1A,B). MV-exposed DC cultures [containing a mixture of infected (GFP+) and uninfected (GFP-) cells] were added to the basolateral side of the tissue models (which were horizontally flipped for this purpose for 60 min and then re-flipped) (Figure 1A). Confirming observations made in similar model systems (27, 39), reconstituted DCs migrated through the tissue model as detected by the presence of DC-SIGN+ cells in close proximity to and within the E-cadherin-positive epithelial layer after 24 h (Figure 1C). This also applied to MV-infected DCs, because MV was transmitted to epithelial cells as evidenced by direct imaging and recovery of GFP+ H358 cells after 4 days (Figure 1D). Further validating the model system, CRISPR-Cas9 mediated ablation of nectin-4, the MV entry receptor on epithelial cells, abrogated acquisition of GFP fluorescence by H358 cells (Figure 1D). Altogether, these findings reveal that our DC reconstituted model system recapitulates MV transmission to airway epithelial cells by these donors and therefore, is suited to dissect important parameters in this process such as dynamic recruitment of donor DCs.

### MV-Infection Promotes Enforced DC Migration in 3D

**Figure 2:**
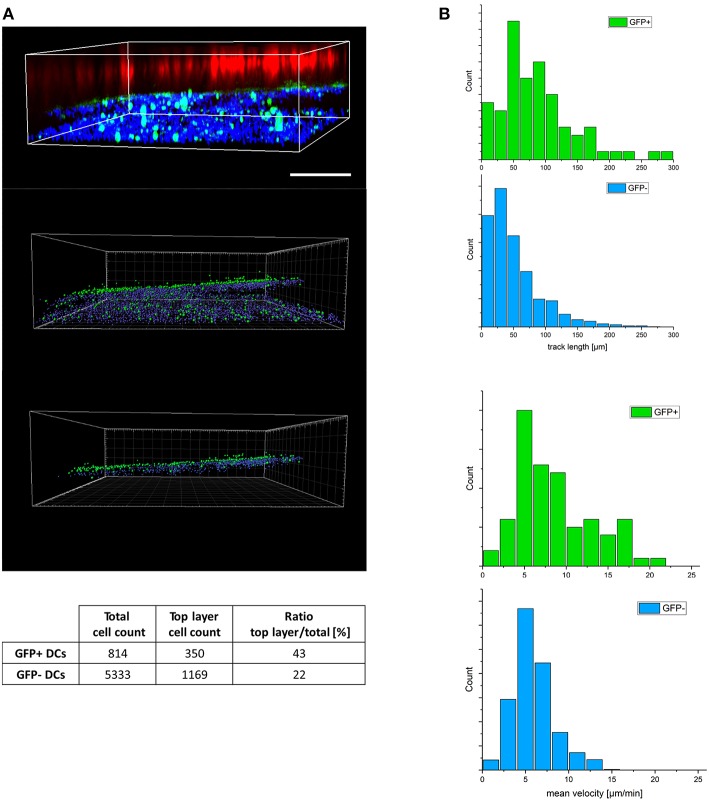
MV-infected DCs reveal enhanced velocity in a 3D environment**. (A)** Z-stacks were imaged from a representative tissue model consisting of eFluor 670 pre-labeled H358 cells (red), fibroblasts (not visible in this plane) and MV-infected DC cultures (pre-labeled with life-DAPI, infected DCs expressing GFP) 2 h following basolateral DC reconstitution. 3D view of raw fluorescence data (upper panel), and after conversion into countable objects via spot finder tool in Imaris (middle panel) and the estimated top layer (bottom panel) as in material and methods section described. To avoid double counting, numbers of GFP+ cells were corrected for the corresponding nuclei count (GFP-). The ratios of the objects in the top layer to the total object count in each group (GFP+/-) was calculated accordingly (table). Scale bar, 200 μm. Data representative of 8 independent experiments. **(B)** Distribution of track length (top panel) and related velocity (bottom panel) for trajectories of GFP +/- DCs extracted from 3D timelapse experiments in 3D models. Representative of three independent experiments.

To efficiently transmit MV in a 3D environment, DCs need to maintain integrity and motility [both of which are subject to MV infected regulation in 2D cultures (22, 23, 38, 40)]. We therefore comparatively analyzed migration of DAPI life stain labeled GFP+ and GFP- DCs in the 3D model containing eFluor 670 pre-labeled H358 cells within 2 h after reconstitution. Imaris-based analysis of object segmented z-stacks allowed for transformation of intensity point clouds into countable objects. As mentioned in methods section, DC migration efficiencies within this time frame could be evaluated by quantification of DAPI-labeled DCs having covered a distance of more than 92 μm toward the H358 layer. Surprisingly, the frequency of migrated GFP+ cells clearly exceeded that of GFP- cells indicating enforced velocity of infected DCs (Figure 2A; Figure S2). 3D time lapse recording of both cell populations in the 3D respiratory model revealed more than twofold higher track lengths and speed of GFP+ DCs as compared to their GFP- counterparts within 2 h (Figure 2B; Figure S3) indicating that DC infection (rather than maturation which was common to both GFP+ and GFP- populations) enhanced DC migration in 3D.

### MV Induces Cytoskeletal Activation in DCs Consistent With a Migratory Phenotype

**Figure 3:**
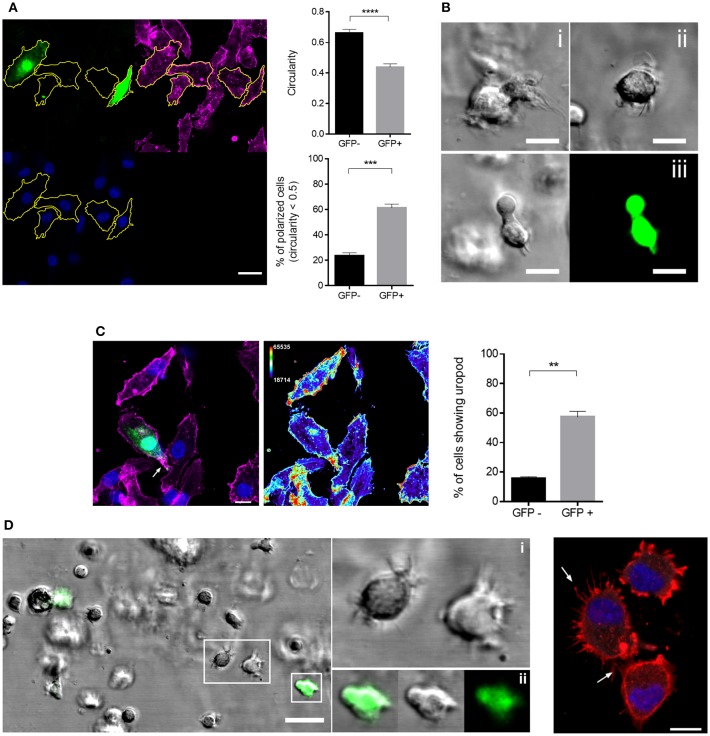
GFP+ DCs polarize more efficiently than GFP- DCs. **(A)** Morphological analysis of GFP+ and GFP- DCs seeded onto FN coated channel slides for 2 h. The manual segmentation strategy for polarity analysis is indicated (left panels; f-actin (phalloidin, magenta), MV-GFP (green), nucleus (blue), scale bar: 20 μm) and circularity values of at least 60 cells in each group (top graph) and percentage of polarized cells, showing circularity index < 0.5 in each group (bottom graph) are shown. Circularity analysis was carried out using ImageJ. Results are shown as mean values ± SEM of four independent experiments. **(B)** Representative images showing the morphology of two different GFP- DCs (i, ii) as differential interference contrast (DIC) and a GFP+ DC (iii) as DIC (left) and fluorescent image (right) embedded in 3D collagen (2 mg/mL) matrix. Scale bar, 10 μm. **(C)** Representative image (left) of uropod detection in DCs, f-actin (phalloidin, magenta), MV-GFP (green), and nuclei (blue). Arrow marks f-actin accumulation at the uropod (left). Heat map of f-actin intensity from the same image (right). The thermal LUT look-up-table (ImageJ) has been adapted using the *Color/Edit LUT* tool. The lowest intensity representing color row has been changed to black in order to obtain a better overall contrast. Scale bar, 10 μm. The graph shows the mean percentage ± SEM of DCs with a well-defined uropod in each group. Data extracted from four independent experiments. **(D)** Representation of DC filopodial structures in 3D collagen (2 mg/ml) (left). Insets show magnification of GFP- DCs with several filopodia (i) and GFP+ DC with a well-defined leading edge less filopodia (ii), scale bar, 20 μm. Right panel: F-actin staining of GFP- DCs (phalloidin, red) and nuclei (DAPI, blue) 2 h after seeding on FN coated channel slides. Arrows mark filopodial structures. Scale bar, 10 μm. ^**^P < 0.01, ^***^P < 0.001, ^****^P < 0.0001.

Acquisition of a polarized morphology associated with actin front-rear translocation is prerequisite to enhanced migration (41). We therefore defined overall cell polarity in MV exposed cultures which for DCs is usually defined by loss of circularity and accumulation of actin and myosin II at the uropod (42, 43). The latter relies on expression of recombinant fluorophore-tagged myosin II which cannot be performed in infected DCs, and uropod markers established for T cells (such as CD43 or pERM) are not established in DCs and fail to do so in our hands (not shown). When they were seeded onto fibronectin (FN), GFP+ cells were significantly less circular than GFP- DCs (Figure 3A, top graph). Indicating a higher degree of polarization of MV-infected DCs, more than 60% of GFP+ DCs revealed circularity levels below 0.5 while this applied to only about 23% of GFP- cells (Figure 3A, bottom graph). Similarly, GFP+ rather than GFP- cells acquired a migratory polarized phenotype also in collagen matrices (Figure 3B; Video S1) suggesting that MV infection indeed enforced cytoskeletal activation in DCs as required for fast migration. Further lending support to this hypothesis, clear segregation of actin-enriched uropods (44) was preferentially observed in FN-seeded GFP+ rather than GFP- cells (Figure 3C), while an overall less polarized phenotype in conjunction with pronounced filopodial structures at the leading edge was seen for GFP- DCs in collagen matrices (Figure 3D).

### Enhanced Migration of MV-DCs in 3D Is Independent of Signals From Epithelial Cells and Amoeboid

**Figure 4:**
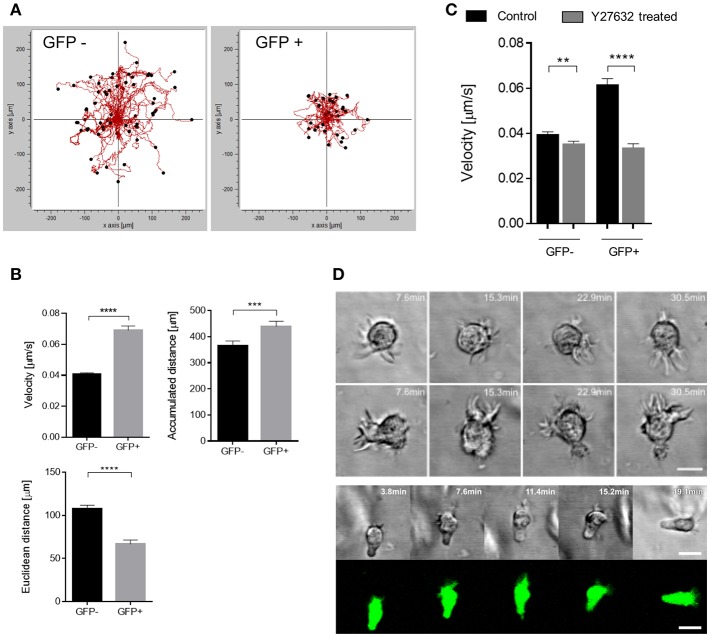
GFP+ cells acquire a fast amoeboid migration mode. **(A)** Representative single cell trajectories graph recorded for 90 min of GFP- (left) and GFP+ (right) DCs embedded in 3D collagen (2 mg/mL) matrices of one data set. **(B)** Quantification of single cell tracks analysis for velocity (left), Euclidean distance (right) and accumulated distance (bottom), (*n* = 7 donors in 6 independent experiments). **(C)** Velocity of single cell tracks of GFP+ and GFP- DCs pre-exposed to Y27632 treated (30 μM) or not (Control) in collagen matrix (2 mg/mL). Graph shows mean ± SEM of three independent experiments each consisting of one donor in three replicates. **(D)** Time-lapse sequence (in minutes) of two different GFP- (top) DCs and a GFP+ (bottom) DC random migration in 3D collagen (2 mg/mL). Scale bars: 10 μm. ^**^P < 0.01, ^***^P < 0.001, ^****^P < 0.0001.

Enforced migration coupled to pronounced polarization of the actin cytoskeleton in infected, GFP+ DCs suggested that these might employ an amoeboid rather than mesenchymal migration mode for fast passage through the 3D environment as described for fast leukocyte migration in complex environments earlier (42, 45). To study virus-induced enforced migration at a mechanistic level, we included collagen matrices for further experiments. These recapitulated features of enhanced GFP+ DC migration seen in the 3D model tissues by confirming their enforced velocity and accumulated distance, while their directionality was inferior to that of GFP-DCs (measured as euclidean distance) (Figures 4A,B). This is retained in the collagen matrix where migration is random and signals potentially provided by H358 cells or fibroblasts in the respiratory models are absent. Further indicating that paracrine signals by H358 cells do not contribute, differential velocity of GFP+ and GFP- DCs was recorded in the absence of H358 cells and fibroblasts (or conditioned media) in collagen coated channel slides and 3D respiratory models (not shown).

In contrast, pre-exposure to the ROCK inhibitor Y27632, known to interfere with amoeboid migration, particularly reduced the velocity of GFP+ DCs, and also targeted that of GFP- DCs to some extent indicating that both populations use this migration mode (Figure 4C). Accumulated distance was also affected to a minor extent for both populations upon ROCK inhibition (Figure S4).

**Figure 5:**
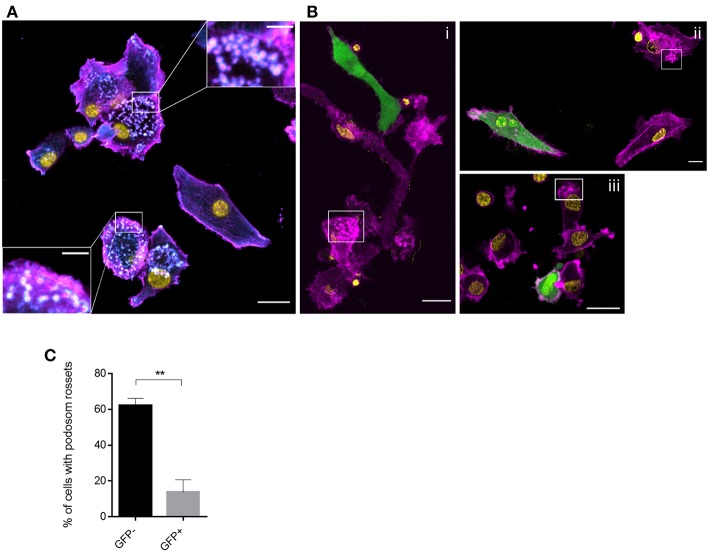
Podosome structures are prevalent in GFP- DCs. Vinculin (cyan), f-actin (magenta), MV (GFP) and nuclei (yellow) were co-detected in MV exposed DCs seeded on FN for 2 h. Overviews are shown in **(A)** and **(B)** (scale bars, 20 μm), insets in **(A)** show magnifications of podosomal structures prominently seen in GFP- cells. In **(B)** boxed regions highlight the podosomal structures which are not present in GFP+ DCs (scale bars, 5 μm). Images are representative for 4 independent experiments. **(C)** The percentage of DCs with podosome rosettes was measured in each group. Graph shows the mean ± SEM of four independent experiments each consisting of one donor. At least 60 cells were analyzed per group. ^**^P < 0.01.

In addition to their higher degree of circularity, frequency and activity of filopodia was found elevated in GFP- DCs (Figures 3A,D, 4D). In contrast, filopodial activity was low in GFP+ DCs (Figure 4D; Video S2) which were also devoid of detectable f-actin+/vinculin+ podosome structures consistent with a more mesenchymal-like type of migration which, in turn, were amply present in GFP- DCs (Figure 5). Altogether this indicates that MV-infection supports a push-and-squeeze fast amoeboid migration mode in DCs while uninfected DCs—though also matured—additionally use the mesenchymal-like mode for random migration in 3D.

### Enforced Migration of Infected DCs Is Supported by the Activity of the SphK/S1P System

**Figure 6:**
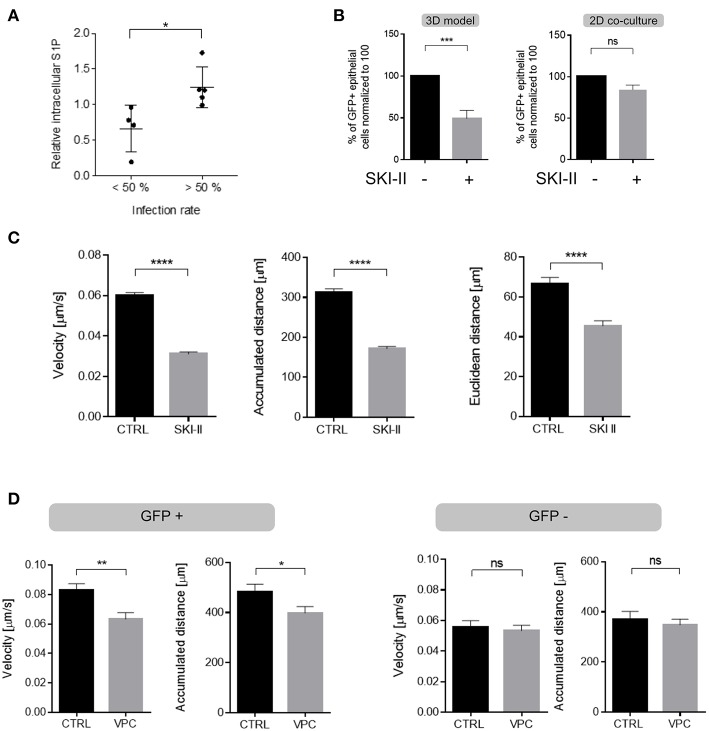
S1P production substantially contributes to enhanced DC velocity. **(A)** Levels of intracellular S1P were determined in MV-infected DC cultures after 24 h (when also infection levels were determined by detection of the viral N protein) and normalized to those obtained for each individual culture exposed to a MOCK preparation (set to 1). Results shown represent each pairwise analyses for cultures revealing infection levels lower (20–49%, *n* = 4) and higher (56–78%, *n* = 5) than 50% with mean values differing significantly (*p* = 0.0159, two tailed Mann-Whitney test) between these two groups. **(B)** MV transmission to H358 cells (shown as % GFP+ H358 cells) from MV-DC cultures treated with SKI-II (10 μM) or not (Ctrl) in 3D tissue models (left) and in 2D co-cultures (right) 4 days after addition of MV-DCs by flow cytometry. Data represents the mean ± SEM of at least three independent experiments, normalized to the untreated control group. **(C)** Quantification of velocity (left), accumulated (middle) and euclidean distances (right) measured in single cell trajectories of GFP+ DCs treated with SKI-II treated (10 μM) or not (CTRL) in 3D collagen (2 mg/mL) for 90 min. **(D)** Quantifications of single cell trajectories of GFP+ (left panel) and GFP- (right panels) DCs treated with VPC (10 μM) or not (CTRL) in 3D collagen (2 mg/mL) for 90 min. Results in **(C,D)** are shown as mean ± SEM of three independent experiments each consisting of one donor in three replicates. **(B–D)** ^*^P < 0.05, ^**^P < 0.01, ^***^P < 0.001, ^****^P < 0.0001.

In common to that of other viruses, MV replication in certain cell lines was found to be enhanced by sphingosine kinases (46), which catalyze production of the bioactive sphingolipid S1P, a potent regulator of cell motility (47, 48). To evaluate whether the SphK/S1P system contributed to enhanced migration of infected DCs, intracellular S1P levels were determined in MV-exposed cultures by LC-MS/MS. When normalized to those measured in MOCK exposed immature DCs, S1P levels produced from high infected cultures (infection levels of more than 50%) significantly exceeded those from low-infection cultures after 24 h (Figure 6A; Figure S6) indicating that S1P may be specifically produced by infected DCs. This could not be directly verified by performing mass spectrometric S1P quantification after sorting which artificially modulates sphingolipid composition nor did a commercially available S1P specific antibody allow for reliable assignment of this low abundant bioactive metabolite at a single cell level or by flow cytometry (not shown). If S1P production was to contribute to enhanced velocity of GFP+ DCs, this should be sensitive to SphK inhibition. A first set of experiments indeed established that the presence of SKI-II (a SphK inhibitor used at a concentration established to be non-toxic for DCs or H358 cells, not shown) reduced transmission of MV to H358 cells in 3D respiratory models (Figure 6B, left panel). Suggesting that the inhibitor acted on DC migration rather than on transmission, the latter was unaffected by SKI-II in DC/H358 co-culture experiments (Figure 6B, right panel), at the same time indicating that in contrast to other cell types, SKI-II may not markedly affect MV replication within 24 h in DCs. Corroborating the importance of SphK activity (and thereby S1P production) in promoting migration of GFP+ DCs, SKI-II pre-treatment substantially reduced velocity of these cells in collagen matrices (Figure 6C), while migration parameters in GFP- DCs were not affected by this inhibitor (Figure S5).

To finally evaluate whether enhancement of DC migration via S1P involves receptor signaling following export, MV-infected cultures were exposed to VPC, an inhibitor blocking signaling via S1P receptor subtypes 1 and 3 (S1PR_1_ and S1PR_3_). VPC exposure selectively reduced velocity of GFP+ DCs and did not detectably affect that of GFP- cells in collagen (Figure 6D). Though not excluding an additional role of intracellular S1P, these findings suggest that S1P receptor signaling acts to promote enforced velocity of infected DCs in an autocrine manner to support their fast amoeboid trafficking through the respiratory epithelial tissue.

## Discussion

3D tissue models integrate host-pathogen interactions and dynamics and are amenable to pharmacologic and genetic intervention which makes them suitable systems to study infection in complex environments and identify targets for intervention. Advancing of an established 3D respiratory tract model allowed us to show that MV infection promotes cytoskeletal activation of DCs resulting in polarization and enhanced velocity thereby favoring transmission to epithelial targets. This did not require paracrine signals provided through fibroblast or epithelial cells, and was rather related to S1P production by infected cells. In supporting DC migration toward and nectin-4 dependent MV transmission to H358 cells (Figures 1B,C), the model recapitulated important parameters of late steps of MV spread. Importantly, transmission efficiencies can be quantified in this tissue model using flow cytometry on recovered H358 cells (Figure 1C). As recovery is not complete and virus-induced fusion does also occur (and fusion cells be lost during recovery or flow cytometry), the frequency of infected epithelial cells is most likely underestimated in our system.

In both the tissue model and collagen 3D systems, MV infection efficiently promoted actin polarization and enhanced velocity in DCs (Figures 2–4). Though less efficiently than TLR4 signaling, MV infection caused phenotypic maturation of immature DCs independently of infection levels (Figure S1), and this may be brought about by production of soluble mediators or ligation of TLR2 (22–24, 36–38). Also in agreement with features resembling LPS-matured DCs, MV-exposed bulk cultures differed from uninfected of Mock-exposed DCs with regard to morphological changes, enhanced motility on FN coated supports and Rac activation (40). In this particular study, responses of the bulk culture were analyzed without considering the impact of direct infection. We now showed that MV-induced cytoskeletal activation in infected DCs was of particular importance for efficient transmission to epithelial cells which may rely on fast migration of donor DCs whose viability is known to be limited upon *in vitro* MV infection (23, 38, 49, 50). Most likely, GFP- DCs also migrate toward epithelial cells, albeit less fast. Slow migration of DCs reconstituted into a respiratory tissue model in the absence of apically added pathogen toward the epithelial layer was reported earlier (27), and we also detected DC-SIGN+ cells there 24 h following addition of uninfected DCs (Figure 1A). Inasmuch virally regulated DC stiffness and deformability contributes to fast migration needs to be determined. MV-infected DCs efficiently squeezed through our 3D models indicating deformability of both the cell body (Figure 2). In contrast, MV transmission by infected macrophages and DCs to epithelial cells seeded onto polyester membranes required a pore size of 3 μm, while uninfected Mo-DCs efficiently passed through a collagen coated 0.4 μm polyester membrane to reach epithelial surfaces (20, 27).

As for most if not all viruses, the actin cytoskeleton was found to be crucial for MV viral entry, replication and particle production mainly in non-hematopoietic cells (19, 51–54). Except for ligation of nectin-4 by MV H protein during entry, MV proteins directly promoting actin cytoskeletal re-organization are unknown. Our findings strongly support that infection triggered activity of the SphK/S1P system efficiently contributes to this process in DCs (Figure 6). Expression of S1P receptor subtypes 1-4 (S1PR_1−4_) has been confirmed in immature and LPS-matured human Mo-DCs (55, 56) and the sensitivity of enforced DC velocity to S1PR_1_ inhibition reveals that this receptor is also expressed and functional on MV-infected DCs (Figure 6D). Not yet shown to occur in these cells, S1PR_1_ signaling can promote F-actin assembly (and notably also vinculin reduction, possibly reflecting podosome dissolution in infected DCs) (57), and both SphK and S1PR_1_ activity can suppress IREα expression and thereby ER-stress mediated pro-apoptotic pathways (58). Induction of NOXA after MV infection and ER stress upon overexpression of the MV glycoproteins in non-hematopoietic cells suggest that MV infection activates this pathway (59–61) and therefore, by triggering SphK/S1PR_1_ system in DCs, MV might both limit ER stress and support DC migration (46). Whether an additional effect of this system on MV replication as described for other cell types also applies to DCs cannot be ruled out (46). If at all, the SKI-II inhibitor marginally affected GFP accumulation in infected DCs (not shown) and MV transmission to co-cultured H358 cells (Figure 6B), yet substantially prevented DC velocity our 3D systems, indicating that the SphK/S1P system may be more important for migration in these post-mitotic cells (Figure 6C). MV mediated regulation of the SphK/S1P system and/or viral and cellular targets in infected cells warrants characterization. Ligation of DC-SIGN by MV (but also specific antibodies or mannan) transient activation of acid sphingomyelinase and thereby, ceramide release which was, however, transient and is not likely to account for S1P accumulation after 24 h (62). MV replication was found sensitive to SphK ablation in cell lines (46) indicating that the enzyme plays a role in this process (interestingly, much less or not in DCs) yet it is not clear from this study whether the virus indeed activates SphK or causes S1P accumulation (which could as well be due to viral interference with expression or function of sphingosine-1-phosphatase or sphingosine-1-phosphate lyase both acting to contract the S1P pool (63). For obvious reasons, viral MV regulation of either of these enzymes will have to rely on established cell systems which, unlike Mo-DCs, allow for standardized high level infection and are easily accessible to genetic modification.

In our model, virus promoted enforced DC velocity independently of paracrine signals provided through fibroblasts and epithelial cells, as it was fully retained in the absence of other cellular components in collagen matrices. As typical for an amoeboid mode of migration, f-actin accumulated at the cell rear and velocity was particularly sensitive to ROCK inhibition (Figures 3, 4) (41, 64). In line with their ability to also use a mesenchymal migration mode, GFP- DCs retained podosome structures and high filopodial activity (Figures 3D, 4D) which is consistent with high chemotactic activity, and progressive slowing down of locomotion with increasing geometrical complexity of the environment (65). High filopodial activity was found to also reflect activity metalloproteases at the tip of these structures which thereby promote directional migration through extracellular matrices (66). This may explain higher directionality in migration by GFP- DCs in the collagen environment (Figures 4 A,B), while GFP+ DCs (revealing reduced filopodia activity) are possibly less efficient at digesting the collagen, and therefore rather employ less directional squeezing for migration through the matrix while keeping their higher speed. Interestingly, DCs not expressing GFP at the time of analysis were able to use both the mesenchymal-like and amoeboid migration mode (Figure 4). It remains unclear inasmuch infection beyond GFP detection levels or differential maturation signals [soluble mediators or TLR2 signaling only rather than infection (36, 37, 67)] contribute to retention or specific acquisition of mesenchymal-like migration.

As employed by us in this study, the 3D respiratory model lacks signals opposing DC peripheral recruitment such as CCL-19 and CCL-20 that would “pull” and thereby counterforce DC outward motility. Because MV-infected cultures fail to upregulate CCR7 and rather retain CCR5 within the time interval of infection studied, the presence of CCR7 ligands is not likely to impact in our system (22), can, however be provided by enhancing the complexity of the model.

This also applies to factors possibly further supporting DC motility. In contrast to similar 3D models where pathogens were added to the apical side of the respiratory epithelial layer (27, 28, 68, 69), epithelial cells in our system were naive with regard to pathogen sensing and this would also apply if primary rather than H358 cells had been used. Therefore, enhancement of directional migration of MV-infected DCs would have to rely on tonic mediators and polarization of the respective receptors to the DC leading edge. Mediators released from bronchial epithelial cells under homeostatic conditions are barely defined. Rather than S1P, these include IL-8, or lipophilic factors which have mainly studied with regard to induction of hypo-responsiveness of epithelial and immune cells in the respiratory tract (70). IL-8 also acts as chemoattractant, and though not shown for epithelial cells, can be induced in response to S1P (71). Therefore, targeted disruption of potentially interesting genes in our H358-Cas9 cells will be informative to evaluate the role of gene products produced constitutively or in response to infected DCs in enhanced MV-DC migration, efficiency of conjugate formation and transmission in a 3D environment in future experiments.

## Data Availability

All datasets generated for this study are included in the manuscript and/or the Supplementary Files.

## Ethics Statement

The use of human material was approved by the ethical committee of the Medical Faculty of the University of Wuerzburg.

## Author Contributions

SD, AK, LJ, FS, and LP did the experiments, MSt, BK, MSa, SS-S, and EA conceived the study and the manuscript, SD, EA, and SS-S wrote the manuscript.

### Conflict of Interest Statement

The authors declare that the research was conducted in the absence of any commercial or financial relationships that could be construed as a potential conflict of interest.
