# Supplementary material for: Measles Virus Infection Fosters Dendritic Cell Motility in a 3D Environment to Enhance Transmission to Target Cells in the Respiratory Epithelium
Source: Front Immunol. 2019 Jun 5;10:1294. doi: 10.3389/fimmu.2019.01294 (PMC6560165; doi:10.3389/fimmu.2019.01294)
Supplement: Supplementary file 1 [file Presentation_1.pptx]

## Slide 1
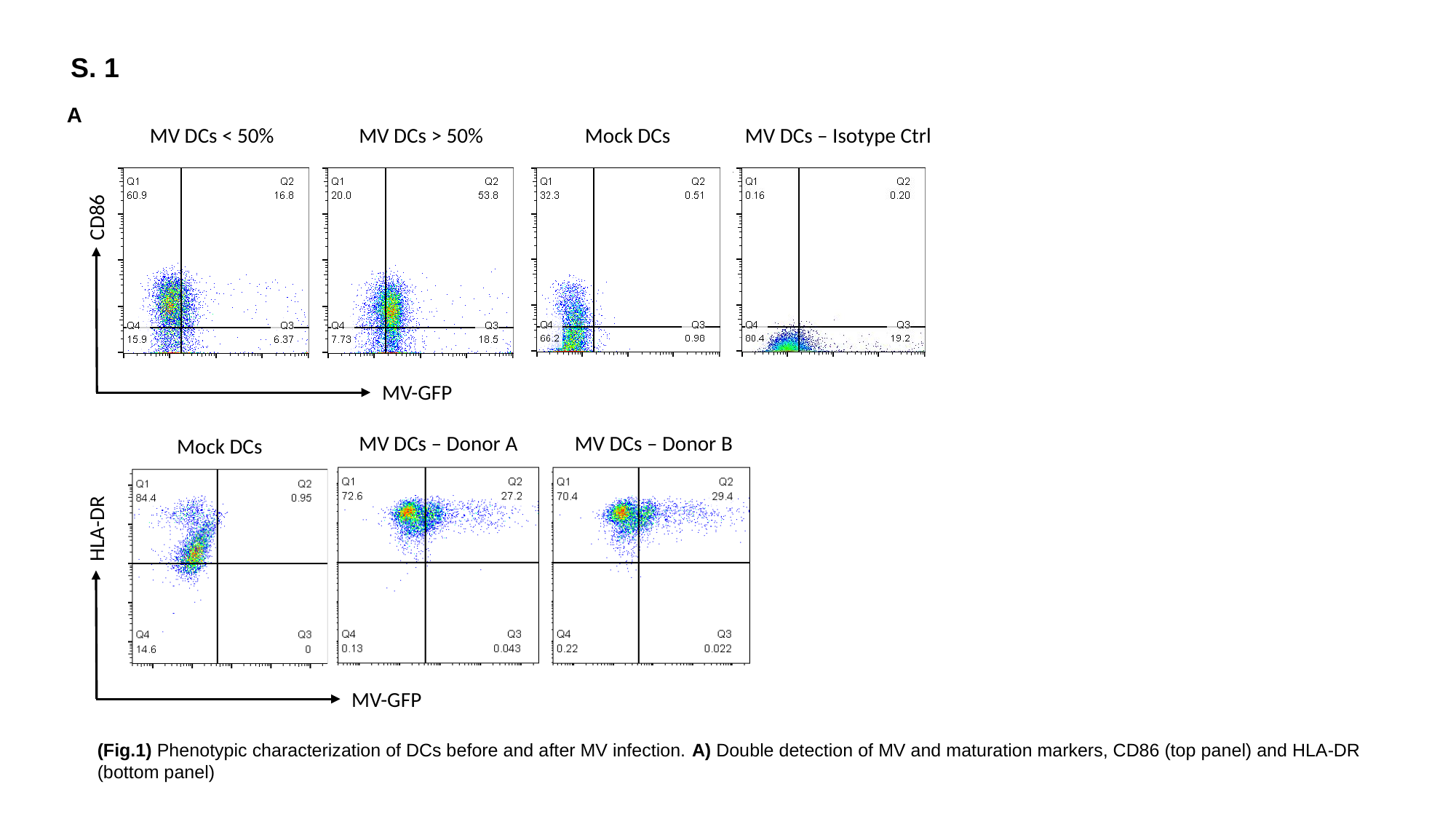

S. 1
A
MV DCs < 50%
MV DCs > 50%
Mock DCs
MV DCs – Isotype Ctrl
CD86
MV-GFP
MV DCs – Donor A
MV DCs – Donor B
Mock DCs
HLA-DR
MV-GFP
(Fig.1) Phenotypic characterization of DCs before and after MV infection. A) Double detection of MV and maturation markers, CD86 (top panel) and HLA-DR (bottom panel)

## Slide 2
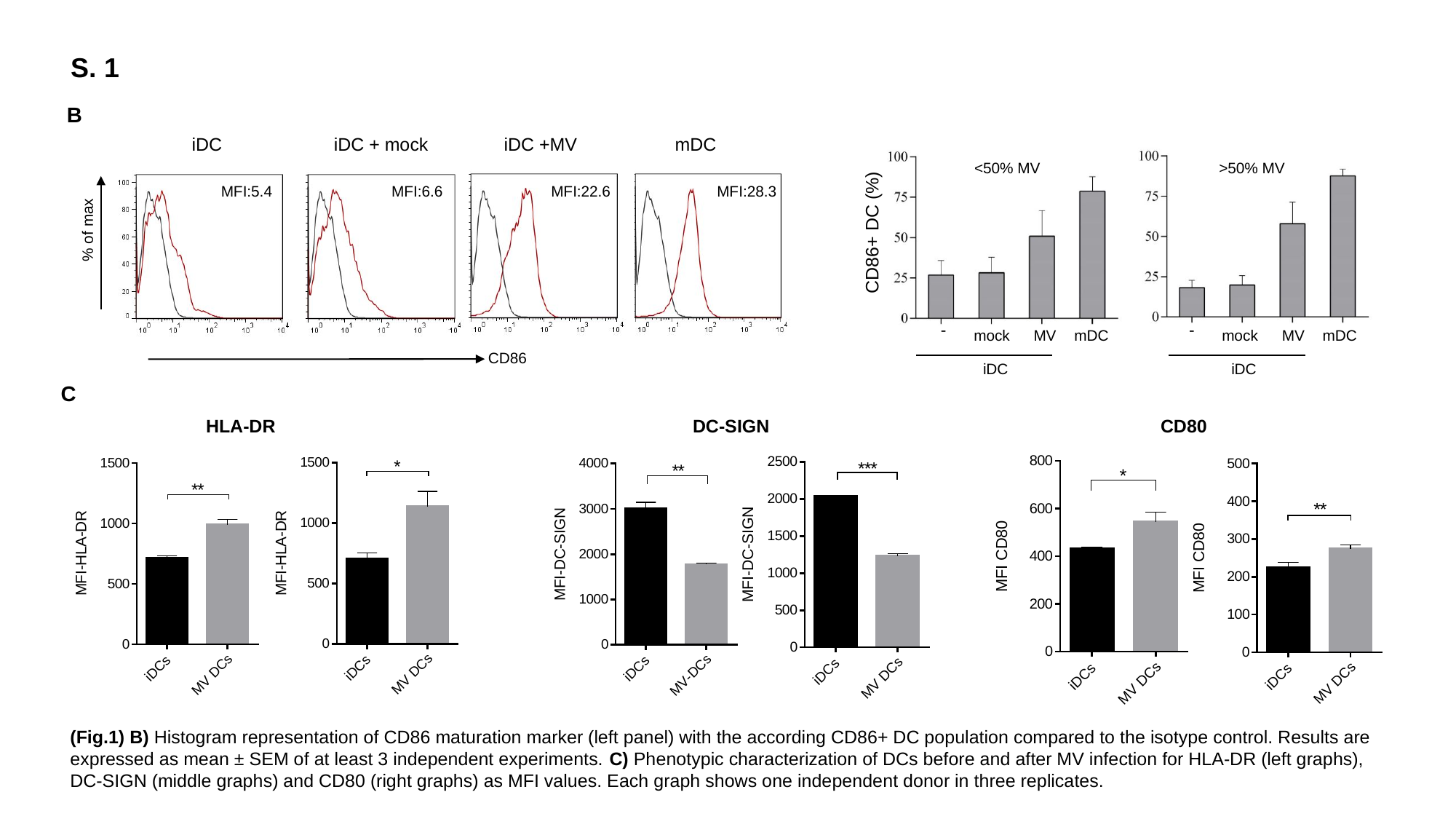

S. 1
B
iDC +MV
mDC
iDC + mock
iDC
MFI:5.4
MFI:6.6
MFI:22.6
MFI:28.3
% of max
CD86
<50% MV
>50% MV
CD86+ DC (%)
-
-
mock
MV
mDC
iDC
mock
MV
mDC
iDC
C
CD80
DC-SIGN
HLA-DR
(Fig.1) B) Histogram representation of CD86 maturation marker (left panel) with the according CD86+ DC population compared to the isotype control. Results are expressed as mean ± SEM of at least 3 independent experiments. C) Phenotypic characterization of DCs before and after MV infection for HLA-DR (left graphs), DC-SIGN (middle graphs) and CD80 (right graphs) as MFI values. Each graph shows one independent donor in three replicates.

## Slide 3
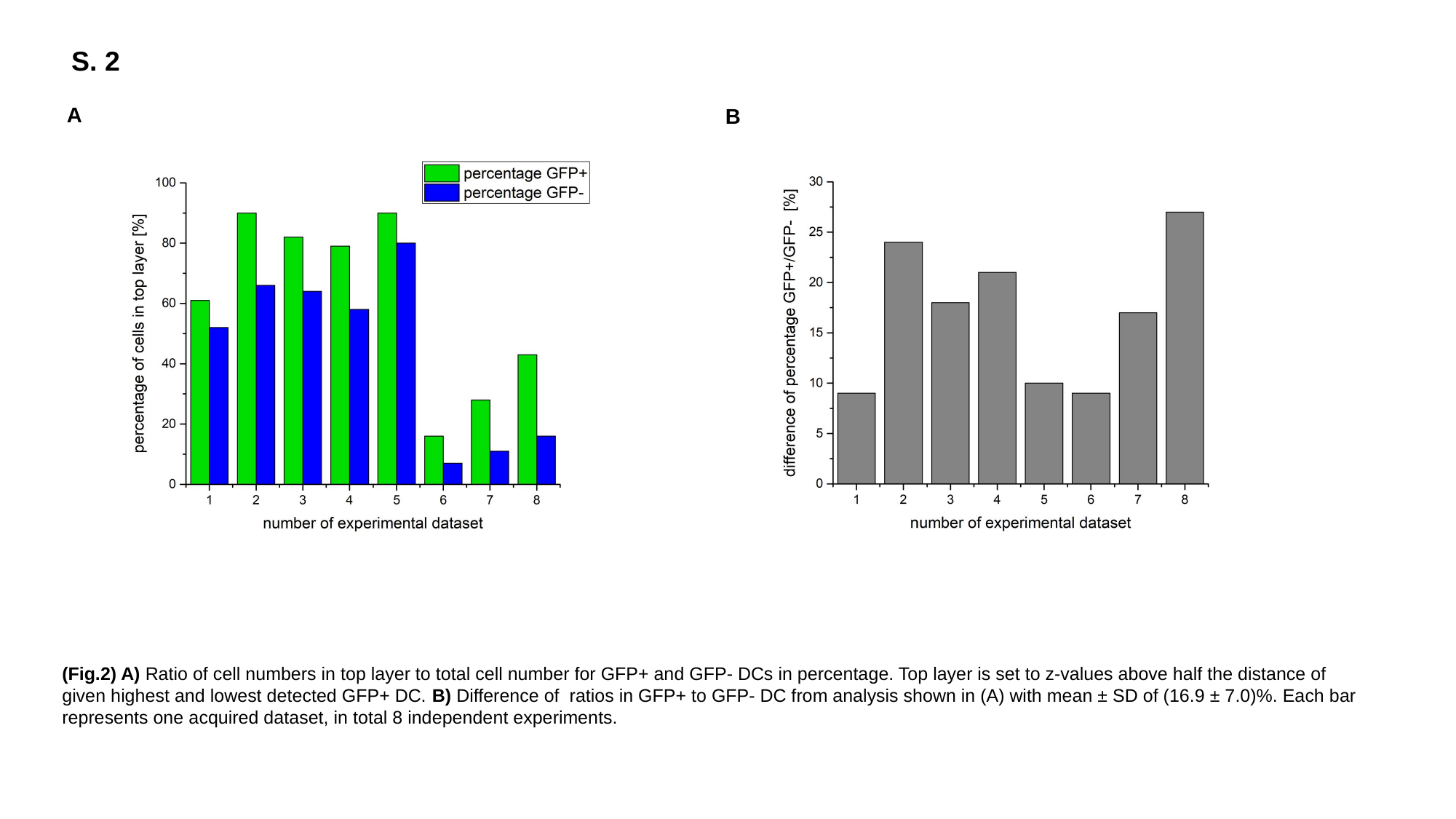

S. 2
A
B
(Fig.2) A) Ratio of cell numbers in top layer to total cell number for GFP+ and GFP- DCs in percentage. Top layer is set to z-values above half the distance of given highest and lowest detected GFP+ DC. B) Difference of ratios in GFP+ to GFP- DC from analysis shown in (A) with mean ± SD of (16.9 ± 7.0)%. Each bar represents one acquired dataset, in total 8 independent experiments.

## Slide 4
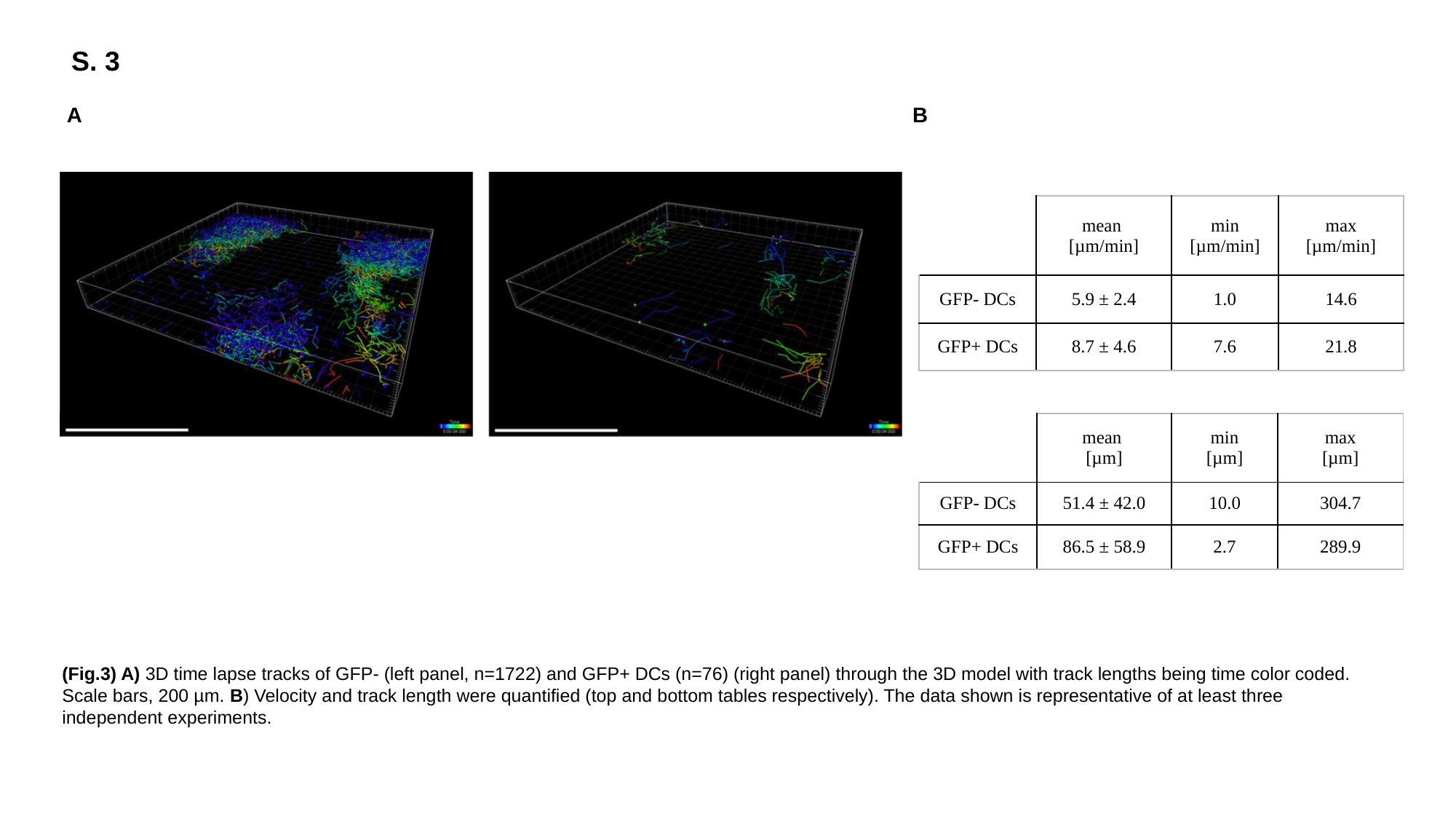

S. 3
A
B
| | mean [µm/min] | min [µm/min] | max [µm/min] |
| --- | --- | --- | --- |
| GFP- DCs | 5.9 ± 2.4 | 1.0 | 14.6 |
| GFP+ DCs | 8.7 ± 4.6 | 7.6 | 21.8 |
| | mean [µm] | min [µm] | max [µm] |
| --- | --- | --- | --- |
| GFP- DCs | 51.4 ± 42.0 | 10.0 | 304.7 |
| GFP+ DCs | 86.5 ± 58.9 | 2.7 | 289.9 |
(Fig.3) A) 3D time lapse tracks of GFP- (left panel, n=1722) and GFP+ DCs (n=76) (right panel) through the 3D model with track lengths being time color coded. Scale bars, 200 µm. B) Velocity and track length were quantified (top and bottom tables respectively). The data shown is representative of at least three independent experiments.

## Slide 5
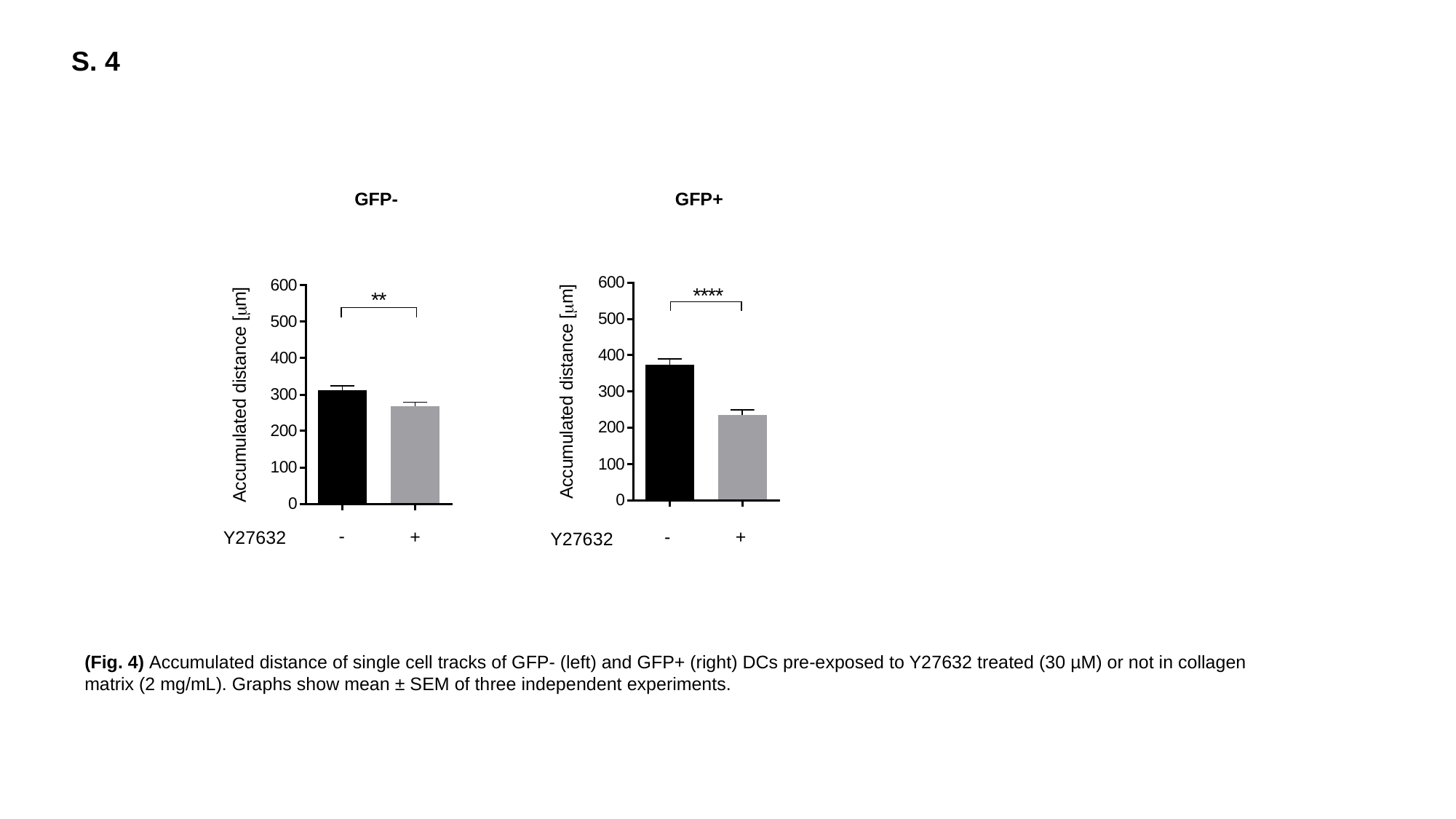

S. 4
GFP-
GFP+
-
+
Y27632
+
-
Y27632
(Fig. 4) Accumulated distance of single cell tracks of GFP- (left) and GFP+ (right) DCs pre-exposed to Y27632 treated (30 µM) or not in collagen matrix (2 mg/mL). Graphs show mean ± SEM of three independent experiments.

## Slide 6
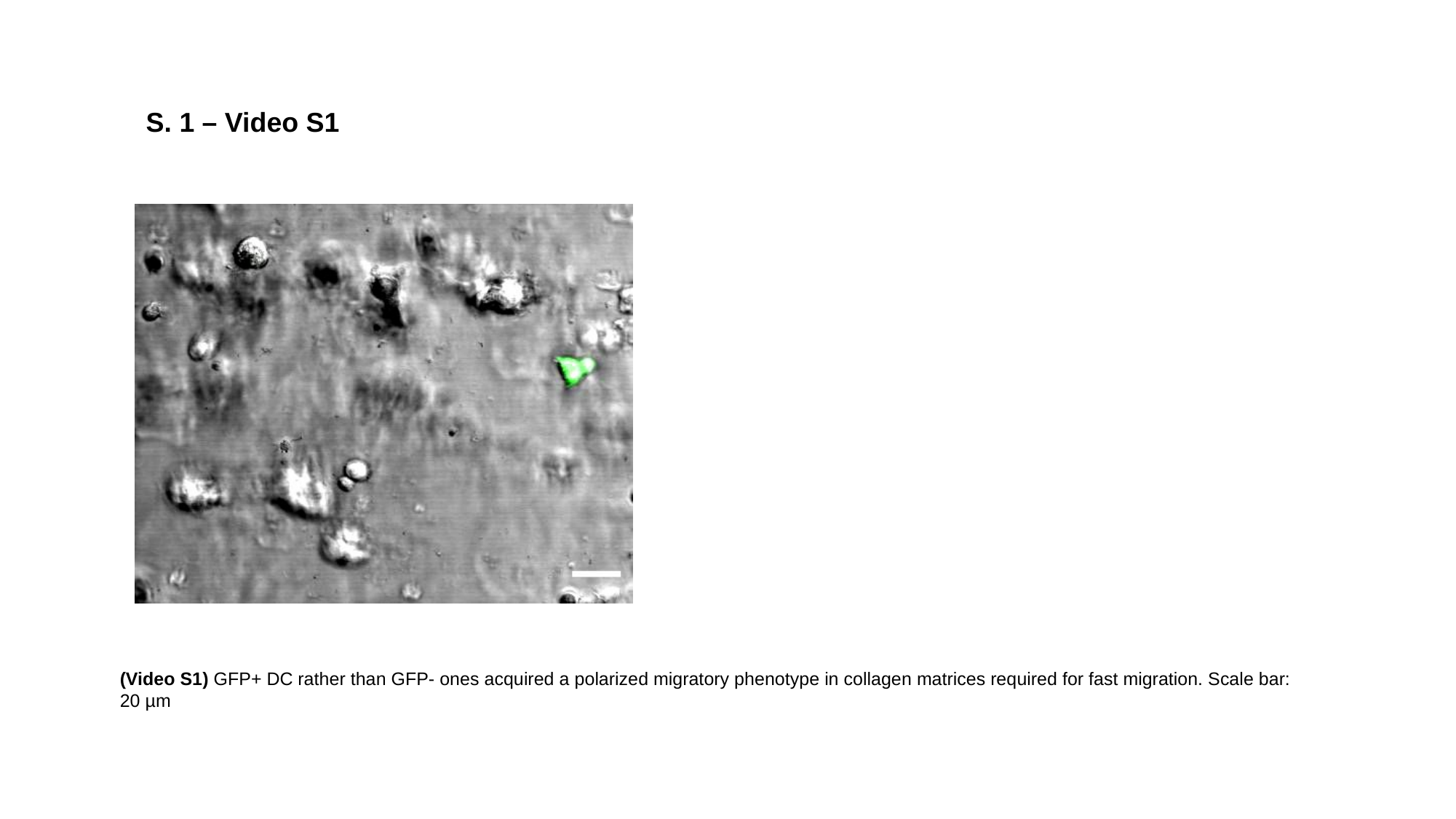

S. 1 – Video S1
(Video S1) GFP+ DC rather than GFP- ones acquired a polarized migratory phenotype in collagen matrices required for fast migration. Scale bar: 20 µm

## Slide 7
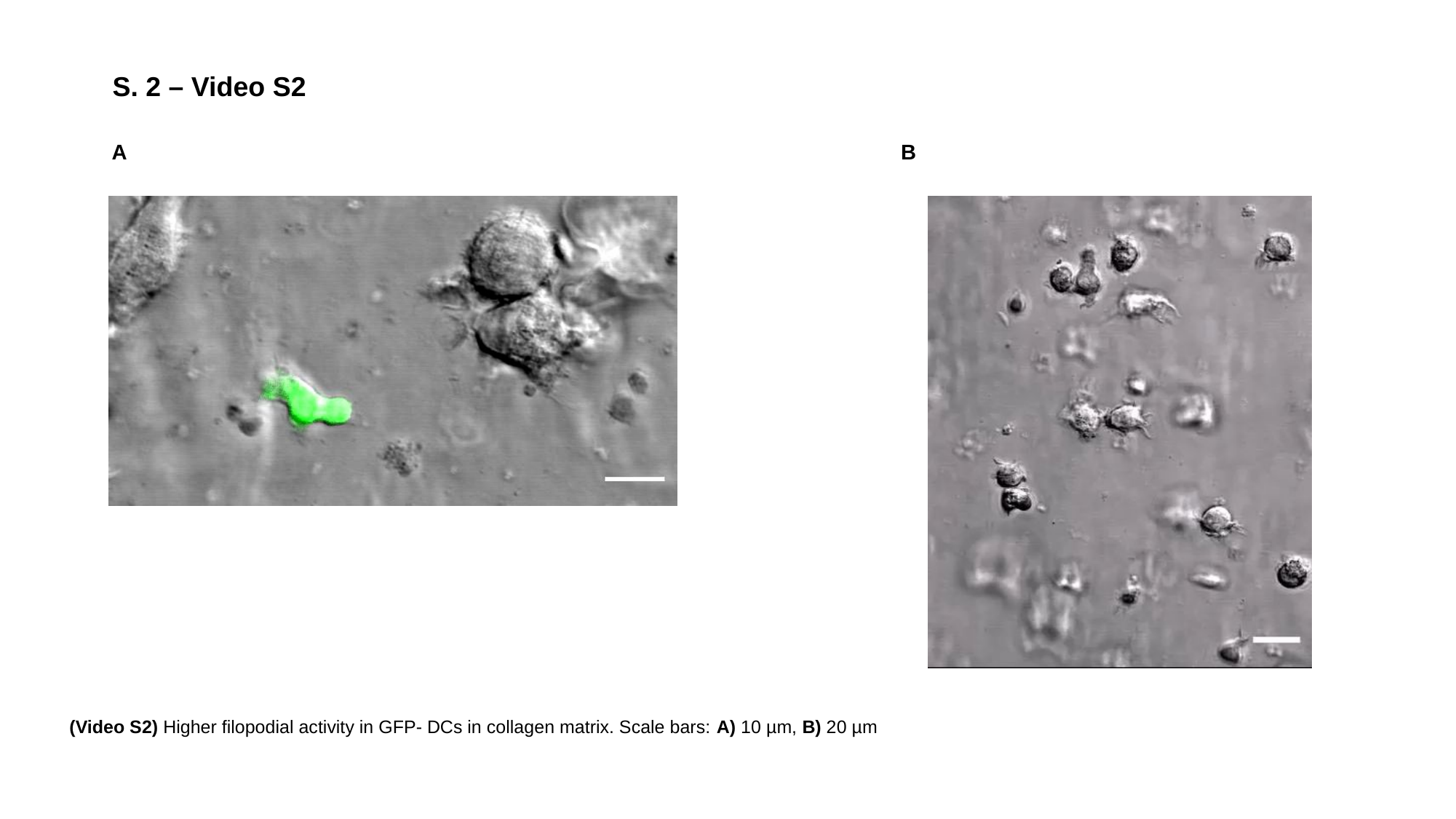

S. 2 – Video S2
B
A
(Video S2) Higher filopodial activity in GFP- DCs in collagen matrix. Scale bars: A) 10 µm, B) 20 µm

## Slide 8
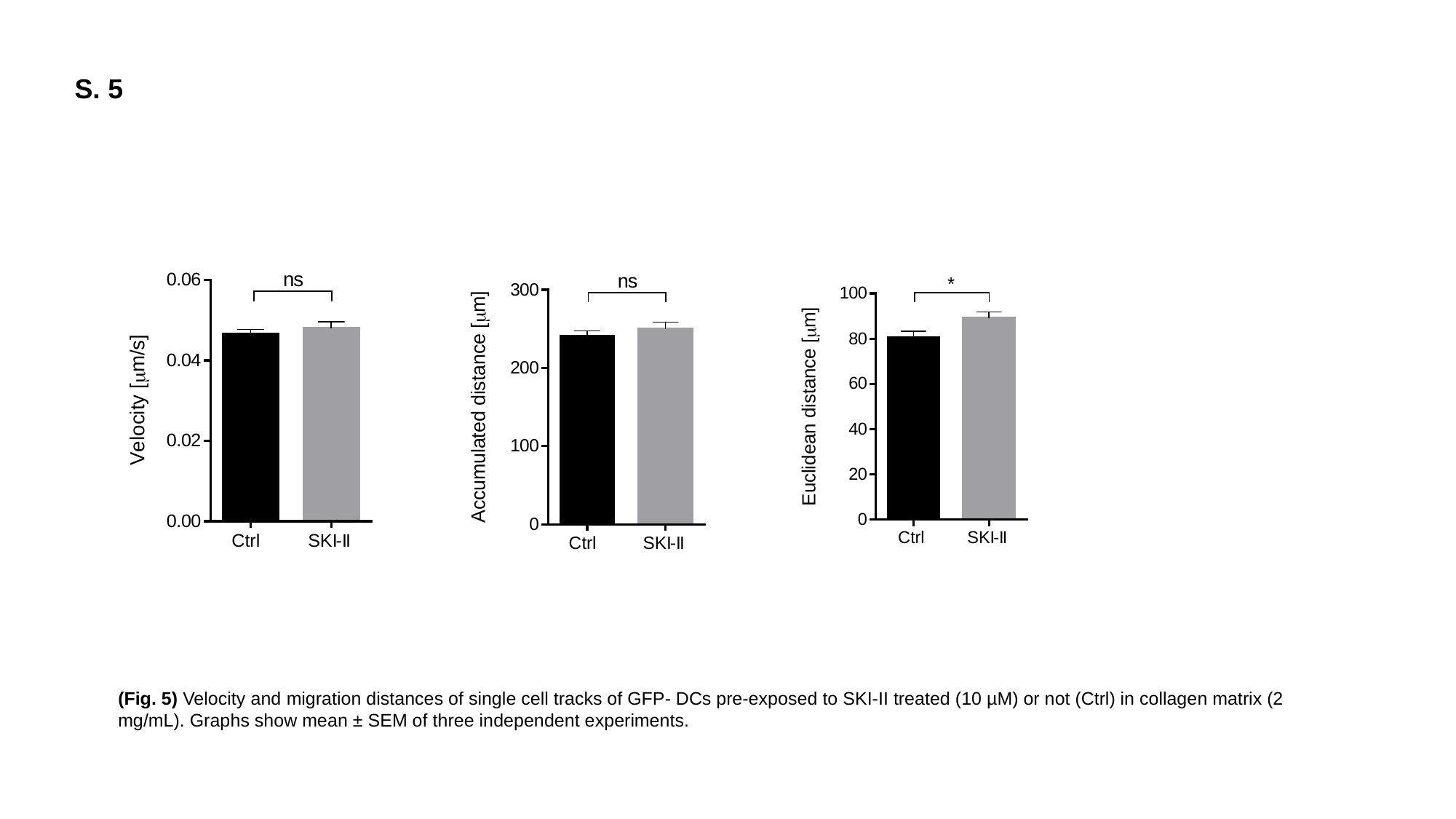

S. 5
(Fig. 5) Velocity and migration distances of single cell tracks of GFP- DCs pre-exposed to SKI-II treated (10 µM) or not (Ctrl) in collagen matrix (2 mg/mL). Graphs show mean ± SEM of three independent experiments.

## Slide 9
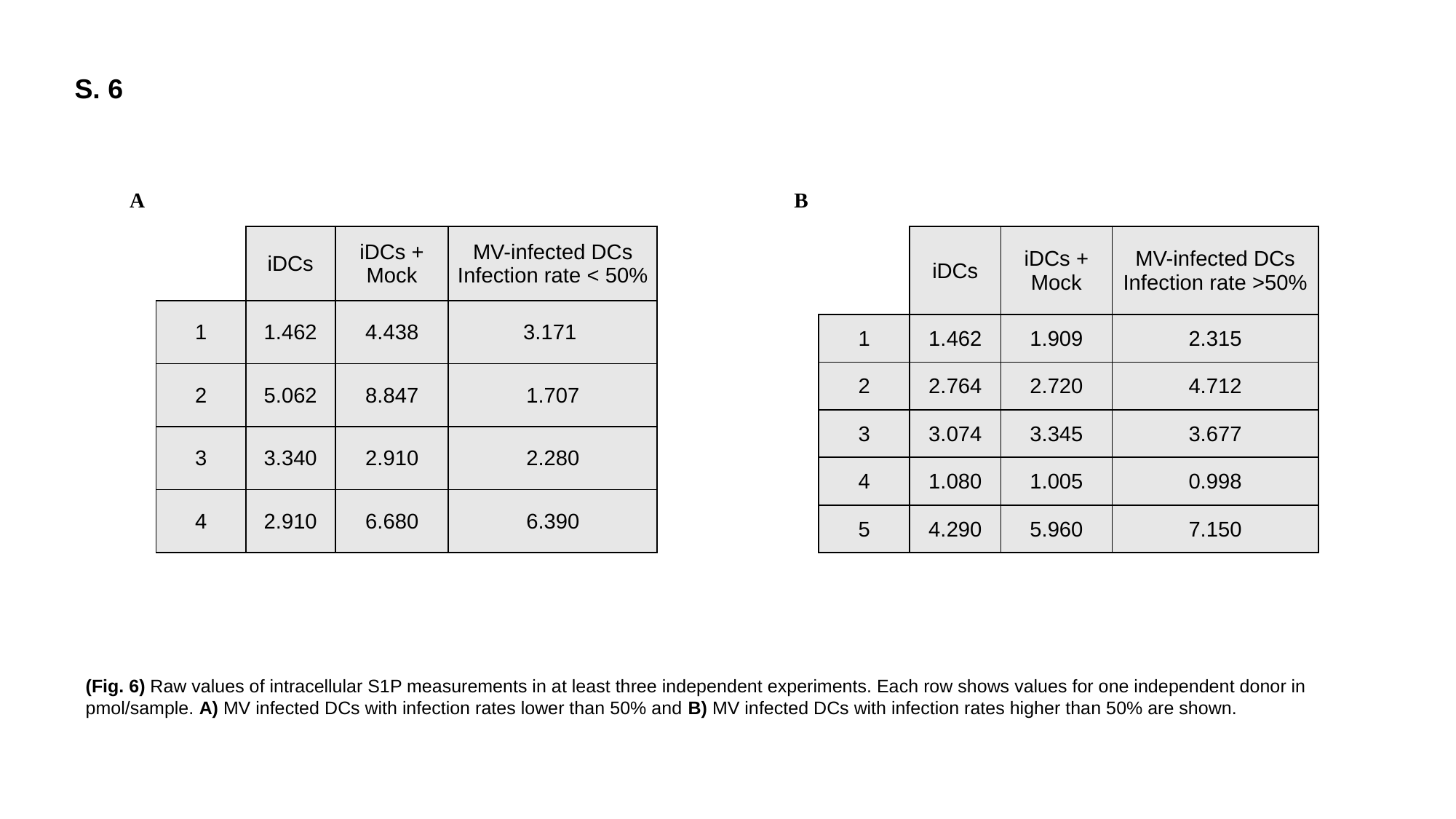

S. 6
B
A
| | iDCs | iDCs + Mock | MV-infected DCs Infection rate < 50% |
| --- | --- | --- | --- |
| 1 | 1.462 | 4.438 | 3.171 |
| 2 | 5.062 | 8.847 | 1.707 |
| 3 | 3.340 | 2.910 | 2.280 |
| 4 | 2.910 | 6.680 | 6.390 |
| | iDCs | iDCs + Mock | MV-infected DCs Infection rate >50% |
| --- | --- | --- | --- |
| 1 | 1.462 | 1.909 | 2.315 |
| 2 | 2.764 | 2.720 | 4.712 |
| 3 | 3.074 | 3.345 | 3.677 |
| 4 | 1.080 | 1.005 | 0.998 |
| 5 | 4.290 | 5.960 | 7.150 |
(Fig. 6) Raw values of intracellular S1P measurements in at least three independent experiments. Each row shows values for one independent donor in pmol/sample. A) MV infected DCs with infection rates lower than 50% and B) MV infected DCs with infection rates higher than 50% are shown.
